# The Epidemiology, Diagnosis, and Cost of Dyspepsia and *Helicobater pylori* Gastritis: A Case–Control Analysis in the Southwestern United States

**DOI:** 10.1111/j.1523-5378.2012.00988.x

**Published:** 2012-09-04

**Authors:** Douglas Mapel, Melissa Roberts, Andrew Overhiser, Andrew Mason

**Affiliations:** * Lovelace Clinic FoundationAlbuquerque, NM, USA; † Northern Arizona GastroenterologyFlagstaff, AZ, USA; ‡ Southwest Gastroenterology AssociatesAlbuquerque, NM, USA

**Keywords:** Dyspepsia, epidemiology, cost, evaluation

## Abstract

**Background:**

Dyspepsia is among the most common complaints evaluated by gastroenterologists, but there are few studies examining its current epidemiology, evaluation, and costs. We examined these issues in a large managed care system in the Southwestern United States.

**Methods:**

We conducted a retrospective case–control analysis of adults with incident dyspepsia or a *Helicobacter pylori*-related condition in years 2006 through 2010 using utilization data. Medical record abstraction of 400 cases was conducted to obtain additional clinical information.

**Results:**

A total of 6989 cases met all inclusion and exclusion criteria. Women had a substantially higher risk of dyspepsia than men (14 per 1000 per year vs 10 per 1000; *p* < .001), and the incidence of dyspepsia increased with age such that persons in their seventh decade had almost twice the risk of those aged 18–29. Hispanic persons had a significantly higher risk of dyspepsia and positive *H. pylori* testing. Dyspepsia cases had a higher prevalence of other chronic comorbidities than their matched controls. Dyspepsia patients had healthcare costs 54% higher than controls even before the diagnosis was made, and costs in the initial diagnostic period were $483 greater per person, but subsequent costs were not greatly affected. Among those aged 55 and younger, the “test and treat” approach was used in 53% and another 18% had an initial esophagogastroduodenoscopy, as compared to 47 and 27%, respectively, among those over the age of 55.

**Conclusions:**

Women and older adults have a higher incidence of dyspepsia than previously appreciated, and Hispanics in this region also have a higher risk. Current guidelines for dyspepsia evaluation are only loosely followed.

Dyspepsia is defined as “chronic or recurrent pain or discomfort centered in the upper abdomen” and is among the most common reasons for visits to primary care providers or referrals to gastroenterology specialists [1–7]. Dyspepsia may be a sign of acute or chronic *Helicobacter pylori* infection, which was shown to be an etiology for dyspepsia after its first identification from stomach biopsy cultures in the early 1980s [8]. Dyspepsia is also a cause of concern for patients over the age of 50 because of the increased incidence of gastric malignancy [9]. Current guidelines for the evaluation and management of dyspepsia emphasize testing for the presence of *H. pylori* infection among persons less than the age of 55 (known as the “test and treat” approach) who do not also have “alarm symptoms” (e.g., weight loss, progressive dysphagia, recurrent vomiting, gastrointestinal bleeding, or a family history of gastrointestinal cancer) and live in a region where the prevalence of *H. pylori* is 10% or greater [10,11]. *H. pylori* eradication therapy is effective in many cases of peptic ulcer disease and resolves symptoms in a substantial proportion with nonulcer dyspepsia [12,13]. It is also presumed that this strategy will reduce the risk of MALT lymphoma and gastric carcinoma, both of which are known to be associated with *H. pylori* infection [14–16]. Guidelines recommend esophagogastroduodenoscopy (EGD) for those presenting with dyspepsia plus alarm symptoms, or who have dyspepsia and are over the age of 55 [10,11].

Even though dyspepsia is a very common clinical complaint and the guidelines for its evaluation and management are well established, there are remarkably few current data on the epidemiology and management of dyspepsia and even fewer data on dyspepsia-related healthcare utilization and costs [17]. Current epidemiologic data could be useful to clinicians to help identify persons at risk of dyspepsia and other *H. pylori*-related complications. Dyspepsia-related utilization data are also needed to compare current clinical practice against the accepted guidelines to identify specific opportunities to improve care. Utilization and cost data could be useful to those who make decisions about allocation of healthcare resources.

The purpose of this study is to describe the current epidemiology, diagnostic evaluation, treatment, healthcare utilization for, and direct medical costs of dyspepsia and *H. pylori* gastritis in one large integrated healthcare system based in the Southwestern United States.

## Methods

This study is a retrospective observational case–control study of adult patients enrolled in the Lovelace Health Plan (LHP), an insurance program operated by Ardent Health Systems who also owns Lovelace Health Systems, a network of hospitals and clinics in New Mexico. The LHP offers a range of employer and self-pay traditional private insurance, health maintenance and preferred provider plans, and Medicaid and Medicare managed care programs. Lovelace Health Systems is based in Albuquerque and is closely affiliated to a multispecialty physician group. Data for this project consisted of LHP administrative and billing data that are routinely abstracted for health services research purposes and were supplemented by data from an electronic medical record (EMR) chart review for 400 randomly selected case patients. Data abstracted from the chart review were used to validate study assumptions about clinical diagnoses and outcomes.

### Case Identification and Control Matching

All adults aged 18 or older who were continuously enrolled in the LHP for at least 12 months during the study period (July 1, 2004 to June 30, 2010) were eligible. The case cohort comprises patients with an outpatient diagnosis of dyspepsia or potentially suffering gastrointestinal distress because of the presence of *H. pylori*. Each case was matched to three control patients who had no utilization for gastrointestinal illnesses.

Cases were identified based on the earliest occurrence of a clinical diagnosis related to dyspepsia or a potentially *H. pylori-*caused condition, utilization of a specific test or procedure for *H. pylori*, or a prescription claim for an indicated *H. pylori* treatment therapy (see Appendix I for specific inclusion criteria). For diagnosis, procedure, or pharmacy events occurring on the same day, patients are classified first according to the diagnosis, and in the absence of a diagnosis code, according to procedure, and in the absence of a procedure, type of pharmacotherapy. The date of the earliest occurrence is the index date for the case. Patients who had a history of upper gastrointestinal cancers or other gastrointestinal disorders that would make management by the usual guidelines inappropriate were excluded (see Appendix I for specific exclusion criteria). The exclusion criteria were revised by the two authors who are experienced gastroenterology physicians to help ensure that the dyspepsia cases and non-dyspepsia controls reflect current clinical practice in this region.

Each case was matched to three non-dyspepsia control patients for the purpose of comparing their utilization and identifying other comorbidities that may be risk factors for *H. pylori*. Controls had at least one episode of similar utilization (outpatient or emergency department (ED) visit) for a non-gastrointestinal complaint. Matching was performed based on the following parameters: sex (exact match), utilization date (within ±6 months of the index date for the case), type utilization (outpatient or ED visit), age at utilization (±2 years as of the index date for the case).

Patients with gastroesophageal reflux disease (GERD) or alarm symptoms were identified using closely related *International Classification of Disease, 9th Revision, Clinical Modification* (ICD-9-CM) codes, including those that matched the alarm symptoms listed in guidelines for use of endoscopy for dyspepsia [18] (Appendix II).

### Study Variables and Analysis

Patient sex, age, and type of health insurance at the time of the index event were derived from administrative records. Hispanic ethnicity was derived using a locally developed and validated software program that assigns ethnicity based on surname. Comorbidities were determined using two methods – the Klabunde/Deyo adaptation of the Charlson index [19] and the Elixhauser method [20]. The Elixhauser method is based on inpatient diagnoses and diagnosis related group (DRG) and was adapted in this study so that outpatient information could be incorporated. Comorbidities were assessed based on ICD-9 diagnosis codes and counted if subjects had an ICD-9 code of interest for a hospitalization, an ED visit or ≥2 outpatient visits. Healthcare utilization (hospitalizations, ED visits, outpatient visits, laboratory visits, and outpatient prescription fills) was derived from the administrative claims. Ambulatory events included laboratory events.

Costs assessed for the study were direct medical costs from the perspective of a health system. All costs are stated as 2009 US dollars. Medical services costs were determined by first applying 2009 Medicare rates for specific CPT/HCPCS codes, and then in the absence of Medicare rates, average actual paid rates (cost for the health plan) adjusted to 2009 costs using the Medical Care Services consumer price index for procedures performed in non-Lovelace facilities. Pharmacy costs were assessed using 80% of published average wholesale price (AWP) for specific medications by National Drug Code (NDC) identifier, multiplied by the prescription quantity dispensed. Pharmacy costs were adjusted to December 2009 prices using the Medical Care Commodities consumer price index. Total direct healthcare costs were the sum of medical services and pharmacy costs. Medical service costs are divided by hospitalization, ED visit, and outpatient visits. For group comparisons, differences were assessed using Pearson's chi-squared test for categorical variables and Student's *t*-test for continuous variables. All statistical tests were two-sided with a level of significance of 0.05 and were conducted with SAS v 9.2 for Windows (SAS Institute, Cary, NC, USA).

## Results

**Figure 1:**
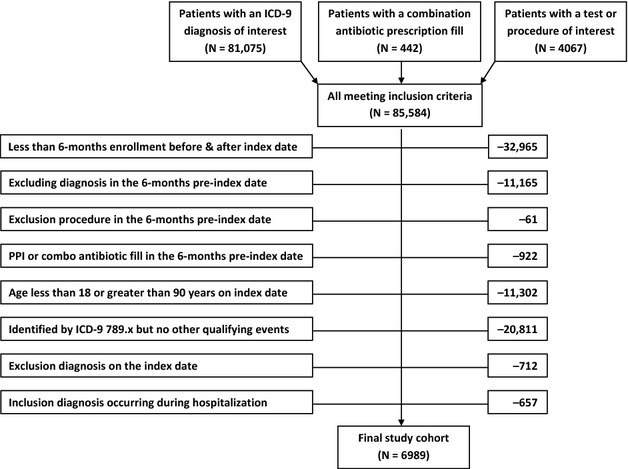
Patients meeting inclusion and exclusion criteria.

A total of 6989 patients met all inclusion and exclusion criteria (Fig. 1). Most cases (86%) met the inclusion criteria by a scheduled outpatient office visit associated with a dyspepsia-related diagnosis, 12% were diagnosed at the time of an urgent care or ED visit, and the remaining 2% were identified only by a prescription fill for an antibiotic combination indicated for *H. pylori* (Table 1). Patients selected for the EMR review were randomly selected from each of the specific categories shown in Table 1. On the basis of EMR information, the index date was confirmed as a new episode for 89% of the diagnosis identified cases; for 5%, there were notes documenting complaints of dyspepsia or gastritis in the 6 months prior to the index date but no associated utilization, and for 6%, there was documentation of dyspepsia or gastritis occurring prior to the index date, but the exact time of the previous occurrence was not provided. Among procedure identified cases, the EMR review found the index date to be the first incidence of dyspepsia for 92%, with 6% having EMR evidence in the baseline period of dyspepsia or gastritis that were not in the claims data, and 3%, EMR notes relating to dyspepsia or gastritis earlier than the index date, but with no specific dates.

**Table 1:** Final case cohort inclusion groups by service location

		Ambulatory visit	ED or urgent care visit	Rx only	Total
Basis for inclusion	Specific category	N = 5994	N = 865	N = 130	N = 6989
Diagnosis a	*Helicobacter pylori* diagnosis	92	9		
	Gastric or duodenal ulcer	302	60		
	Dyspepsia	592	71		
	Other GI diagnosis	1427	244		
	GI signs or symptoms	2666	465		
Diagnosis identified cases					5928
Procedure	EGD	26	0		
	*H. pylori-*related test	839	10		
Procedure identified cases					885
Prescription	≥2 *H. pylori* antibiotics	40	6	130	
Prescription identified cases					176

ED, emergency department; EGD, esophago-gastro-duodenoscopy, GI, gastrointestinal.

a Subjects categorized in the order shown; thus, the 302 subjects with an ulcer diagnosis did not have an *H. pylori* diagnosis, the 592 with dyspepsia did not have an ulcer diagnosis or *H. pylori* diagnosis, etc.

**Figure 2:**
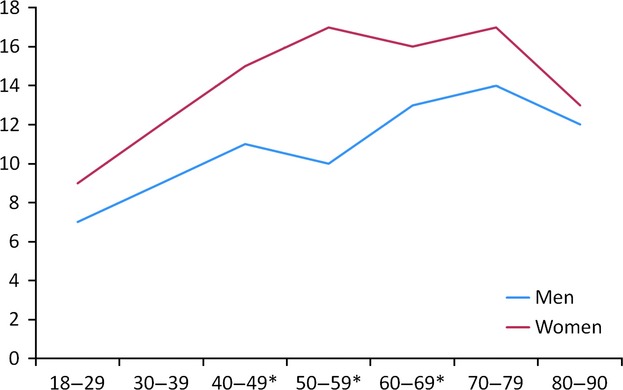
Annual incidence of dyspepsia (cases per 1000) by age and gender. *p Value for difference between women and men in this age group <.05.

In this cohort, women were substantially more likely to have dyspepsia than men (Table 2 and Fig. 2). Across the 5-year study period, women averaged 14 incident cases per 1000 per year, while men averaged 10 per 1000 per year (*p* < .001). The mean age for the cohort was 52, but the age-adjusted incidence of dyspepsia was lowest among younger adults and increased with age until a peak in the seventh decade (Fig. 2). Dyspepsia cases were slightly more likely to be Hispanic (43.6%) than their age- and gender-matched controls (39.9%), and cases were also slightly more likely to be enrolled in Medicare or Medicaid than their matched controls (Table 2). In the EMR review, we found that of the 81 men who had some form of *H. pylori* testing, 36% were positive, as compared to 24% of the 157 women tested (*p* = .06). Of the 49 Hispanic persons who had a *H. pylori* test, 41% tested positive, as compared to 17% of 103 non-Hispanic white people (*p* < .01).

**Table 2:** Baseline demographic characteristics for dyspepsia case and control cohorts

	Case	Control^a^	*p* Value^b^
No. of patients	6989	20967	
Age, mean (SD)	52.5 (18.4)	52.4 (18.6)	0.71
Age categories, N (column %)			
Age 18–39	1811 (26)	5510 (26)	0.55
Age 40–55	2011 (29)	6112 (29)	0.55
Age 56–70	1774 (25)	5180 (25)	0.26
Age > 70	1393 (20)	4165 (20)	0.90
Gender, N (column %)			
Women	4476 (64)	13428 (64)	1.00
Men	2513 (36)	7539 (36)	
Hispanic	3049 (44)	8369 (40)	<0.0001
Insurance^c^, N (column %)			
Commercial	4619 (66)	15371 (73)	<0.0001
Medicare	1403 (20)	3894 (19)	0.0055
Medicaid	1025 (15)	1757 (8)	<0.0001

a Cases matched to controls based on gender, age (±2 years), type of index event, index month/year (within ±6 months).

b Chi-square test for frequencies; Student's *t*-test for means.

c Individuals may have >1 type of insurance.

Other non-gastrointestinal illnesses (comorbidities) were much more common among persons with dyspepsia than among their matched controls (Table 3). Among dyspepsia cases, 29.8% had one or more other significant comorbidities as compared to 18.1% of controls (*p* < .001). The Charlson index, which is a prognostic score based on serious comorbidities that affect short-term survival, was increased in 10.7% of cases as compared to 6.2% of controls (Table 3). Hypertension, diabetes, and chronic obstructive pulmonary disease (COPD) were the most common chronic conditions among both cases and controls and increased by more than 50% among cases. However, the highest odds (greater than 2.0) were seen for COPD, drug abuse, fluid and electrolyte disorders, obesity, and rheumatoid arthritis/collagen vascular diseases. Deficiency anemias, such as iron and vitamin B12 deficiency, were three times more common among cases (Table 3).

**Table 3:** Baseline comorbidities among dyspepsia case and control cohorts

Comorbidity assessment^a^	Case	Control^b^	*p* Value^c^
No. of patients	6989	20967	
Charlson Comorbidity Index^d^, N (%)			
Charlson = 0	6239 (89.3)	19676 (93.8)	<0.001
Charlson = 1	587 (8.4)	1026 (4.9)	<0.001
Charlson ≥ 2	163 (2.3)	265 (1.3)	<0.001
Elixhauser comorbidities (adapted)^e^, N (%)			
Alcohol abuse	46 (0.7)	62 (0.3)	<0.001
Chronic blood loss anemia	7 (0.1)	32 (0.2)	0.31
Chronic obstructive pulmonary. disease	328 (4.7)	415 (2.0)	<0.001
Coagulopathy	13 (0.2)	34 (0.2)	0.67
Congestive heart failure	59 (0.8)	103 (0.5)	<0.001
Deficiency anemias	169 (2.4)	170 (0.8)	<0.001
Depression	174 (2.5)	279 (1.3)	<0.001
Diabetes (with chronic complications)	87 (1.2)	145 (0.7)	<0.001
Diabetes (without chronic complications)	508 (7.3)	992 (4.7)	<0.001
Drug abuse	42 (0.6)	46 (0.2)	<0.001
Fluid and electrolyte disorders	62 (0.9)	65 (0.3)	<0.001
Hypertension	851 (12.2)	1555 (7.4)	<0.001
Hypothyroidism	223 (3.2)	361 (1.7)	<0.001
Liver disease	9 (0.1)	13 (0.1)	0.08
Lymphoma	10 (0.1)	22 (0.1)	0.41
Metastatic cancer	7 (0.1)	35 (0.2)	0.21
Obesity	119 (1.7)	163 (0.8)	<0.001
Other neurological disorders	102 (1.5)	179 (0.9)	<0.001
Paralysis	17 (0.2)	17 (0.1)	<0.001
Peripheral vascular disease	56 (0.8)	86 (0.4)	<0.001
Psychoses	199 (2.8)	229 (1.1)	<0.001
Pulmonary circulation disease	22 (0.3)	63 (0.3)	0.85
Renal failure	34 (0.5)	70 (0.3)	0.07
Rheumatoid arth/collagen vasc dz	127 (1.8)	162 (0.8)	<0.001
Solid tumor withoutmetastasis	91 (1.3)	296 (1.4)	0.50
Valvular disease	54 (0.8)	121 (0.6)	0.07
Weight loss	32 (0.5)	21 (0.1)	<0.001
Any Elixhauser comorbidity	2084 (29.8)	3787 (18.1)	<0.001

a Only comorbidities with >10 patients are shown.

b Cases matched to controls based on gender, age (±2 years), index month/year (within ±6 months).

c Chi-square test for frequencies; Student's *t*-test for means.

d Based on 6 months prior to index date; Note that neither case nor control patients had ulcer diagnoses (one of the Charlson comorbidities) as patients with pre-index ulcers were excluded.

e Based on ICD-9-CM diagnoses associated with any emergency department visit or hospitalization, or ≥2 outpatient visits.

Correspondingly, dyspepsia patients had higher utilization of medical services in every category in the 6 months before they were diagnosed or treated for dyspepsia or *H. pylori* (Table 4). Not surprisingly, dyspepsia cases had more prescription fills for a non-steroidal anti-inflammatory drug (NSAID) at baseline than controls (17.9 vs 10.5%), although NSAID use is undoubtedly understated for both groups because of undocumented over the counter utilization. Among cases included in the EMR review, 19% had claims evidence of NSAID use, and another 10% had evidence of NSAID or COX-2 inhibitor use based on EMR records. From the index date through the first 7 days of the follow-up periods, dyspepsia cases averaged $1091 for visits and procedures and $54 for treatment, while matched controls averaged $625 and $36, respectively. Healthcare costs in the remainder of the follow-up period were substantially increased from those in the 6 months before diagnosis, although the ratio of total direct costs for cases to controls was approximately the same, 1.5:1, in the baseline period and from 7 days through 6 months post-index.

**Table 4:** Healthcare utilization for dyspepsia case and control cohorts during the 6-month baseline and follow-up periods and the 8-day index period

	Baseline^a^		Index + 7 days^a^		Follow-up (after 7 days)^a^	
			
	Case	Control^b^	Case	Control^b^	Case	Control^b^
Healthcare utilization patients with ≥1 visit (% of cohort)						
Ambulatory visits	84.4	65.1	91.2	93.2	93.1	83.2
ED visits	11.2	4.0	13.8	11.2	16.1	8.6
Hospitalizations	4.5	3.0	2.0	1.3	10.7	7.8
Hospitalizations >1 day	3.5	2.4	1.6	0.8	7.9	6.1
Patients with ≥1 prescription claim (% of cohort)						
NSAID	17.9	10.5	1.5	1.8	16.1	12.6
Cox-2 inhibitors	1.2	0.9	0.2	0.1	1.1	0.7
Healthcare utilization rate (visits per 100 persons)						
Ambulatory visits	517	327	140	125	767	557
ED visits	17	5	16	12	26	12
Hospitalizations (any)	7	5	2	2	19	14
Hospitalizations >1 day	4	3	2	1	10	7
Costs (US$ per person, mean)						
Outpatient services	$1625	$1106	$635	$373	$3025	$1912
ED services	$251	$83	$358	$196	$483	$196
Hospitalizations	$272	$178	$97	$57	$728	$548
Subtotal – medical services	$2148	$1367	$1091	$625	$4235	$2656
Pharmacy	$837	$576	$54	$36	$1166	$751
Total direct cost	$2985	$1943	$1144	$661	$5401	$3407
Ratio total direct cost (case/control)	1.54		1.73		1.59	

AWP, average wholesale price; ED, emergency department; NSAID, non-steroidal anti-inflammatory drug; SD, standard deviation.

a All differences between cases and controls were significant (*p* < .001), based on chi-square test for frequencies and proportions, and Student's t-test for means except for NSAID and Cox-2 inhibitor use for index + 7 days (*p* > .05), and any hospitalization for index + 7 days (*p* = .002).

b Cases matched to controls based on gender, age (±2 years), index month/year (within ±6 months); cases = 6989, controls = 20,967.

The diagnostic testing at the time of initial diagnosis was stratified by those aged 55 and younger versus those over the age of 55 to assess overall compliance with guidelines. Among patients aged 55 and younger, 53% had some form of *H. pylori* diagnostic testing, and another 18% had an EGD at the time of initial diagnosis (Table 5). The presence of alarm or GERD symptoms had only a modest effect on the likelihood of obtaining an EGD: 32% of patients with alarm symptoms and 24% of those with GERD had an EGD, as compared to 14% with no additional documented symptoms.

**Table 5:** Occurrence of EGD, *Helicobacter pylori* testing, or *H. pylori* treatment by age category and evidence of alarm symptoms or GERD at the time of initial diagnosis

	Total		Alarm Sx		GERD, no Alarm Sx		No Alarm Sx or GERD	
				
	N	%	N	%	N	%	N	%
*Age ≤55 years*								
Total cases	3822		600		331		2891	
EGD								
EGD with biopsy	578	15^a^	169	28^a^	69	21	340	12^a^
Other diagnostic EGD	129	3^a^	25	4^a^	13	4	91	3^b^
Any EGD	682	18^a^	189	32^a^	80	24	413	14^a^
*H. pylori* test/treatment								
*H. pylori* test	2025	53^a^	282	47^a^	212	64	1531	53^a^
*H. pylori* treatment	613	16^b^	93	16	63	19	457	16^b^
*H. pylori* test AND treatment	366	10^a^	59	10	50	15	257	9^a^
*H. pylori* test OR treatment	2272	59^a^	316	53^a^	225	68	1731	60^a^
*Age > 55 years*								
Total cases	3167		594		248		2325	
EGD								
EGD with biopsy	716	23^a^	283	48^a^	65	26	368	16^a^
Other diagnostic EGD	203	6^a^	81	14^a^	17	7	105	5^b^
Any EGD	865	27^a^	339	57^a^	78	32	448	19^a^
*H. pylori* test/treatment								
*H. pylori* test	1479	47^a^	199	34^a^	154	62	1126	48^a^
*H. pylori* treatment	570	18^b^	96	16	47	19	427	18^b^
*H. pylori* test AND treatment	382	12^a^	55	9	40	16	287	12^a^
*H. pylori* test OR treatment	1667	53^a^	240	40^a^	161	65	1266	55^a^

EGD, esophago-gastro-duodenoscopy; GERD, gastroesophageal reflux disease; Alarm Sx, alarm symptoms.

a Significant difference between age categories for measure, *p* < .01.

b Significant difference between age categories for measure, *p* < .05.

More patients over the age of 55 had an EGD than those under 55 (27 vs 18%; *p* < .01), but the proportion was still low given the guideline recommendation that all over the age of 55 with dyspepsia have an EGD (Table 5). Patients over the age of 55 were only slightly less likely to have a *H. pylori* test (47 vs 53%; P < 0.1), and the “test and treat” approach was about the same (16 vs 18%; P < 0.5). However, the majority of those who had documented alarm symptoms (57%) did have an EGD, and those who had GERD were more likely to have an EGD than those without alarm symptoms or GERD (32 vs 19%; *p* < .01). In the EMR review, we found that of the 143 persons aged 55 and under who had a *H. pylori* test, 17% had a positive result, and that of the 171 persons over the age of 55 who had a *H. pylori* test, 27% had a positive result (*p* < .05).

## Discussion

Our analysis found that dyspepsia and *H. pylori*-related gastrointestinal diagnoses were remarkably common among adults in this health system, with an annual incidence of 13 per 1000. A unique finding was that women were substantially more likely to have dyspepsia than men, especially in the fifth decade. The incidence among adults increased with age until the fifth decade in women and the seventh decade in men. Dyspepsia patients had a substantially higher prevalence of chronic comorbidities as compared to their age- and gender-matched controls, especially in conditions such as COPD and alcohol abuse that are associated with cigarette smoking or other exposures that also can cause gastritis. Some conditions such as deficiency anemias were substantially increased even prior to the diagnosis of dyspepsia. However, over two-thirds of dyspepsia patients had no other active chronic illness at the time of initial diagnosis. Healthcare utilization and direct medical cost are higher among dyspepsia patients, but after accounting for the costs directly related to dyspepsia evaluation and management, the proportional difference in direct healthcare costs between cases and controls was not subsequently affected. The test and treat approach was found in slightly more than half of the patients under the age of 55. However, contrary to current guidelines, only 27% of persons aged 55 and over had an EGD. Although the value of alarm symptoms as an indication for testing is a controversial issue [21], the presence of alarm symptoms did appear to modestly affect EGD utilization. This population-based assessment suggests that the increased risk of dyspepsia among women and older adults merits additional investigation.

Although review articles and guidelines often note that dyspepsia is a common complaint, there are few studies that have examined the epidemiology of dyspepsia. El-Serag et al. [22] conducted a systematic review of international studies published between 1976 and 2002 that reported population-based surveys of the prevalence of dyspepsia. The survey methods were highly variable and confounded by GERD symptoms, but when the definition of dyspepsia is restricted to upper abdominal pain, lifetime estimates ranged from 5 to 12% worldwide. There is a great degree of overlap between dyspepsia and GERD, making examination of the epidemiology of either complaint somewhat confounded. A review of studies that examined the overlap between dyspepsia and GERD by Gerson et al. [23] found that 38% (range, 21–63%) of patients with GERD also report dyspepsia symptoms. They also found that patients with dyspepsia were at risk of subsequent development of GERD, and that those with both dyspepsia and GERD had substantially worse health-related quality of life scores as compared to just GERD alone. The American Gastroenterological Association guidelines for the evaluation and management of dyspepsia suggest that those initially presenting with both dyspepsia and GERD complaints be managed as GERD [10,11], but we found that those with both diagnoses were evaluated more aggressively than those with dyspepsia alone.

We were surprised to find that approximately two-thirds of dyspepsia patients were women, with much of the difference between men and women occurring at middle age. The American Gastroenterological Association guidelines do not discuss sex as a risk factor for dyspepsia. In their review of 22 dyspepsia studies, El-Serag et al. found that six reported a slightly higher incidence of dyspepsia among women (range, 10–30% greater), three found a higher incidence among men (range, 5–10% greater), and the rest did not report any differences [22]. Data from other surveys suggest that there may be differences by sex in presenting gastrointestinal symptoms or gastritis complications. In a comparison of the first diagnosis of gastrointestinal complaints among adults aged 18–65 enrolled in US managed care plans, women comprised 61% of 14,593 patients with nonulcer dyspepsia, as compared to 53% of 3456 patients with peptic ulcer disease, 54% of 36,233 with gastroesophageal reflux, and 66% of 44,129 patients with abdominal signs and symptoms of undetermined cause [24]. In their analysis of the Healthcare Cost and Utilization Project Nationwide Inpatient Sample, which captures a 20% stratified sample of all hospitalizations in the United States, Wang et al. reported that 46.5% in 1993 and 49.1% in 2006 admitted with a primary diagnosis of peptic ulcer (ICD-9-CM 531–533) were women, suggesting that there is no gender difference in the complication of ulceration with bleeding and perforation [25]. Gastric cancer continues to have a male-to-female ratio of 2:1, although the incidence of gastric cancer continues to decline especially among men [9]. The prevalence of sero-positive *H. pylori* infection among adults participating in the Third National Health and Nutrition Survey in the United States was slightly higher among men (34%) than women (31%) [26]. In our EMR review, we found that men were more likely to have a positive *H. pylori* test, although we are reluctant to draw conclusions from this observation because of the limitations of the study design. It is possible that gender-based differences in healthcare seeking behavior within this system could also explain our observed differences, but we do not have evidence from this or other studies in this system to support that theory.

Age was also an important risk factor for dyspepsia in our cohort, with persons in the seventh decade having twice the risk of young adults. Remarkably few epidemiologic studies have examined age as a risk factor for dyspepsia [22], and guidelines do not identify age as a dyspepsia risk factor [10,11]. The prevalence of *H. pylori* infection is known to increase with age, and surveys have found that a majority over the age of 60 in the United States are colonized, but the recommendation that all persons aged 55 and older have EGD is based on concern for malignancies, not the higher risk of *H. pylori* [27,28]. A higher proportion of persons over the age of 55 in our EMR review who were tested for *H. pylori* had a positive result (27 vs 17%) but additional studies are needed to see whether this accurately reflects the true *H. pylori* infection rate in our population. The revised National Institute for Clinical Excellence dyspepsia guidelines from the UK do not support routine EGD for dyspepsia at any age, but they do encourage urgent referral for endoscopy for patients over the age of 55 who have unexplained and persistent recent onset dyspepsia [29]. Our analysis found that persons over the age of 55 were only slightly less likely to have some form of *H. pylori* testing than those aged 55 and younger (47 vs 53%), but more likely to have an EGD (27 vs 18%). Perhaps this result should not be too surprising: in a recent survey of gastroenterologists and primary care providers, only 27% of general internal medicine doctors and 36% of nurse practitioners agreed with this guideline [30]. Randomized clinical trials for dyspepsia treatment have usually excluded persons aged 65 and older [31], and cost-effectiveness studies on dyspepsia management ignore age as a factor even though elderly patients have much higher average total medical costs and non-gastrointestinal comorbidities [32].

In our review of the literature, we could not find any articles presenting actual direct medical cost data for dyspepsia patients who were treated within the last decade. In a study of 1987 US National Medical Expenditure Survey, dyspepsia patients were shown to be heavy users of healthcare resources, averaging 14 visits and US$3850 in overall healthcare charges per patient per year [33]. Actual cost data for dyspepsia evaluation and management are needed for more accurate assessments of the cost-benefits of various approaches. While *H. pylori* eradication therapy is highly cost effective for peptic ulcer disease, the cost effectiveness of *H. pylori* eradication therapy in nonulcer dyspepsia is less certain [34]. Cost-effectiveness studies to date tend to be based on estimated costs for various services, and their results are heavily dependent on the actual costs for the more expensive diagnostic procedures [35]. Current cost-effectiveness studies also tend to ignore the impact of age and comorbidities in their calculations, but our analysis reveals that these factors are not inconsequential.

There are limitations to our study that should be considered. Our case identification methods are based on a system using billing and procedure codes that may have missed or misclassified some dyspepsia patients. Our EMR review was able to validate that over 90% of the cases were incident cases of dyspepsia, and the rest were re-exacerbations of chronic symptoms, but it is still possible that some patients were misclassified by our case identification system. In a study that examined the validity of a similar system used to identify *H. pylori* patients, Thirumurthi et al. [36] found that the positive predictive value (PPV) for the ICD-9-CM diagnostic code 041.86 was 97.4% in the outpatient setting, but that triple eradication drug therapy had a PPV of only 73.7%. Another limitation is that our results represent the practice patterns of physicians included in one managed care system, and their choices are undoubtedly affected by the availability of specific services such as the urea breath test, which might be more accessible in other systems. The majority of dyspepsia patients in our cohort are managed by primary care providers, and a recent survey has demonstrated that they may be less specific than gastroenterologist in distinguishing dyspepsia from GERD or irritable bowel syndrome [30].

Our analysis is based on provider data, and given that patients have access to over-the-counter proton-pump inhibitors and other home treatments for GERD and dyspepsia, one can assume that those who seek medical attention for these symptoms are a selected population. However, even with the growth of over-the-counter treatments and reduction in the population prevalence of *H. pylori* infection, our analysis confirms that dyspepsia continues to be a very common clinical complaint and has a costly impact on the healthcare system.

## Appendix I

### Inclusion Criteria for Cases

1. Adults aged 18–90 years who are continuously enrolled in the health plan for at least 12 months are eligible.
2. Encounter (inpatient, emergency department (ED), or outpatient) with one of the following *International Classification of Disease, 9th Revision, Clinical Modification* (ICD-9-CM) diagnosis codes:

**Table:**

Code	Description
041.86	*Helicobacter pylori* (*H. pylori)*
531.x	Gastric ulcer
532.x	Duodenal ulcer
533.x	Peptic ulcer
534.x	Gastrojejunal ulcer
535.00–535.61	Other gastrointestinal codes related to *H. pylori*
536.8	Dyspepsia and other specific disorders of function of the stomach
536.9	Unspecified functional dyspepsia
789.x	Abdominal signs and symptoms of undetermined cause

**3.** Evidence of one of the following CPT procedure codes:

**Table:**

Code	Description
43239	Upper gastrointestinal endoscopy (EGD) with biopsy
78267	Urea breath test, C-14 (isotopic); acquisition for analysis
78268	Urea breath test, C-14 (isotopic); analysis
83009	*H. pylori*, blood test analysis for urease activity, non-radioactive isotope (e.g., C-13)
83013	*H. pylori*; breath test analysis for urease activity, non-radioactive isotope (e.g., C-13)
83014	*H. pylori*; drug administration
86677	Antibody; *H. pylori*
87338	Infectious agent antigen detection by enzyme immunoassay technique, qualitative or semiquantitative, multiple-step method; *H. pylori*, stool
87339	Infectious agent antigen detection by enzyme immunoassay technique, qualitative or semiquantitative, multiple-step method; *H. pylori*

**4.** Patients who do not meet any of the diagnosis or procedure criteria may also be selected who have evidence of one of the following pharmaceutical claim:
  - Prescription for a prepackaged eradication therapy (Prevpac, Helidac, Pylera)
  - Prescriptions (same day claims) for a pair of antibiotics commonly used to treat *H. pylori* (e.g., clarithromycin and amoxicillin) (note that this should also capture triple and quadruple therapies)

Prescriptions for above were identified based on NDC code. Specific NDC codes were identified using the following methodology:

- For commonly used treatments:
  - Pull NDC codes and related information for AHFS classes 081212 Macrolide (for clarithromycin), 081216 Penicillin (for amoxicillin), 084000 Anti-infective (for metronidazole), and 081224 Tetracylcine (for tetracycline).
  - Review generic and brand names and form of NDC codes.
  - Extract NDC codes for oral forms of amoxicillin, clarithromycin, tetracycline, and metronidazole.
- For prepackaged eradication therapies:
  - Pull NDC codes and related information for AHFS classes 564000 Miscellaneous GI drugs and 81228 Miscellaneous Combination Agents Antibacterials.
  - Review generic and brand names and form of NDC codes.
  - Extract NDC codes for brand names Prevpac, Helidac, and Pylera.

### Exclusion Criteria for Cases

To help ensure that the cases are free of substantive gastrointestinal complaints,

1. Patients could not have met one of the inclusionary criteria in the 6 months prior to being identified as a case.
2. Patients must have been enrolled in the health plan for at least 6 months prior case identification and have no evidence of any of the following gastrointestinal-related ICD-9-CM diagnoses during the 6-month pre-diagnosis period:

**Table:**

Code	Description
001–009	Intestinal infectious diseases [only applies to 1 month prior to index date]
150–159	Malignant neoplasm of digestive organs and peritoneum
195.2	Malignant neoplasm abdomen
197.4	Secondary malignant neoplasm of small intestine including duodenum
230.x	Carcinoma in situ of digestive organs
235.5	Neoplasm of uncertain behavior of other and unspecified digestive organs
307.5x	Other and unspecified disorders of eating
452	Portal vein thrombosis
456.0–456.21	Esophageal varices
520–579	Diseases of the digestive system
643	Excessive vomiting in pregnancy
747.61	Gastrointestinal vessel anomaly [bleeding]
750.3	Tracheoesophageal fistula esophageal atresia and stenosis
750.4	Other specified anomalies of esophagus
750.7	Other specified anomalies of stomach
935.1–935.2	Foreign body in esophagus, stomach
938	Foreign body in digestive system unspecified
983.0–983.9	Toxic effect of corrosive aromatics, acids, and caustic alkalis
V10.03–V10.04	Personal history of malignant neoplasm of esophagus or stomach
V44, V55	Artificial opening status, openings requiring attention/ management

**3.** Patients must not have had one of the following diagnostic EGD procedure performed during the 6-month pre-diagnosis period:

**Table:**

Code	Description
43200	Esophagus endoscopy
43202	Esophagus endoscopy, biopsy
43234	Upper gastrointestinal endoscopy, examination
43235	Upper gastrointestinal endoscopy, diagnosis
43237	Endoscopic ultrasound examination, esophagus
43238	Upper gastrointestinal endoscopy w/ultrasound fine needle biopsy
43242	Upper gastrointestinal endoscopy w/ultrasound fine needle biopsy
43259	Endoscopic ultrasound examination

**4.** Patients must not have evidence in claims of a prescription filled for a proton pump inhibitor (PPI) in the 6-month period prior to the index date. Prescriptions for PPI were identified based on NDC code. Specific NDC codes were identified using the following methodology:
  AHFS class 562836 (proton pump inhibitors) or where brand/generic contained esomeprazole, lansoprazole, omeprazole, pantoprazole, rabeprazole.
**5.** Patients identified based on inclusionary diagnosis or procedure events must have been seen in an ambulatory setting (ED or outpatient facility).

### Inclusion Criteria for Controls

Adults aged 18–90 years who are continuously enrolled in the health plan for at least 12 months are eligible.

### Exclusion Criteria for Controls (at the Point in Time When Matched to Cases)

1. Cannot meet any of the inclusion criteria for cases.
2. Same exclusion criteria as for cases.
3. Have no evidence of a personal history of digestive system diseases (V12.70-V12.79) during the 6-month pre-diagnosis period
4. Patients must not have had a procedure related to the esophagus or stomach performed during the 6-month pre-diagnosis period (CPT codes 43xxx).

## Appendix II

### Evidence of Use of Non-Steroidal Anti-Inflammatory Drugs (NSAIDs)/Cox-2 Inhibitors

The use of NSAIDs or Cox-2 inhibitors (in baseline period or from the index date forward) was assessed. NDC codes (and associated generic and brand names) for NSAIDs and Cox-2 Selective NSAIDs for Healthcare Effectiveness Data and Information Set (HEDIS) 2009 measures were used to identify use.

### Evidence of Gastroesophageal Reflux Disease (GERD) or Gastrointestinal Alarm (ALARM) Symptoms

Evidence of GERD or ALARM symptoms was based on ICD-9-CM diagnosis codes appearing in claims for the index date and the 6 month period prior to the index date. For ALARM, ICD-9-CM diagnosis codes were identified used alarm symptoms described by Ikenberry et al. [18]. Codes used were as follows:

**Table:**

GERD	
Code	Description
530.11	Reflux esophagitis^a^
530.81	Esophageal reflux^a^
787.1	Heartburn

a For diagnosis/procedure/prescription identified cases, ICD-9-CM could not appear 6 months before index; for procedure/prescription identified cases, ICD-9-CM also not on index date.

**Table:**

ALARM	
Code	Description
280.0	Iron deficiency anemia secondary to chronic blood loss
280.1	Iron deficiency anemia secondary to inadequate dietary iron intake
280.8	Other specified iron deficiency anemias
280.9	Unspecified iron deficiency anemia
281.0	Pernicious anemia
281.1	Other vitamin B12 deficiency anemia
281.2	Folate-deficiency anemia
281.9	Unspecified deficiency anemia
285.9	Unspecified anemia
438.82	Dysphagia due to cerebrovascular disease
531.0	Acute gastric ulcer w/hemorrhage^a^
531.4	Chronic or unspecified gastric ulcer w/hemorrhage^a^
531.6	Chronic or unspecified gastric ulcer w/hemorrhage and perforation^a^
532.0	Acute duodenal ulcer with hemorrhage^a^
532.2	Acute duodenal ulcer with hemorrhage & perforation^a^
532.4	Chronic or unspecified duodenal ulcer with hemorrhage^a^
533.0	Acute peptic ulcer with hemorrhage^a^
533.2	Acute peptic ulcer with hemorrhage & perforation^a^
533.4	Chronic or unspecified peptic ulcer with hemorrhage^a^
534.0	Acute gastrojejunal ulcer with hemorrhage^a^
534.4	Chronic or unspecified gastrojejunal ulcer with hemorrhage^a^
535.01	Acute gastritis with hemorrhage^a^
535.11	Atrophic gastritis with hemorrhage^a^
535.21	Gastric mucosal hypertrophy with hemorrhage^a^
535.31	Alcoholic gastritis with hemorrhage^a^
535.41	Other specified gastritis with hemorrhage^a^
535.51	Unspecified gastritis & gastroduodenitis with hemorrhage^a^
535.61	Duodenitis with hemorrhage^a^
536.2	Persistent vomiting^b^
578.0	Hematemesis^b^
578.1	Blood in stool^b^
578.9	Unspecified hemorrhage of gastrointestinal tract^b^
783.21	Loss of weight
787.01	Nausea with vomiting
787.03	Vomiting alone
787.20	Dysphagia, unspecified
787.22	Dysphagia, oropharyngeal phase
787.23	Dysphagia, pharyngeal phase
787.24	Dysphagia, pharyngoesophageal
787.29	Other dysphagia
789.3x	Abdominal or pelvic swelling, mass, or lump^a^
793.4	Nonspecific abnormal findings on radiological & other examination of the gastrointestinal tract
V16.0	Familial history of malignant neoplasm of gastrointestinal tract

a ICD-9-CM part of inclusionary diagnosis criteria.

b For diagnosis/procedure/prescription identified cases, ICD-9-CM could not appear 6 months before index; for procedure/prescription identified cases, ICD-9-CM also not on index date.
